# A rare case report: an inextricable shoulder pain as the exclusive presentation of lung adenocarcinoma with metastasis over contralateral clavicle

**DOI:** 10.1186/s40001-021-00493-y

**Published:** 2021-02-27

**Authors:** Karl Wu, Yu-Hao Huang

**Affiliations:** 1 Department of Orthopedic Surgery, Far Eastern Memorial Hospital, New Taipei City, Taiwan, ROC; 2 Department of Materials and Textiles, Oriental Institute of Technology, New Taipei City, Taiwan, ROC

**Keywords:** Clavicular metastasis, Lung adenocarcinoma, Sites of metastasis

## Abstract

**Background:**

Lung cancer is the fourth most common form of the tumor spreading to the bone. Among all patients of lung carcinoma, the most common sites of bone metastasis are vertebrae, ribs, and pelvis. By comparison, the clavicle is an extremely rare site of metastases not only in the population of lung cancers but among all types of tumors. Enlightened by this existing fact, we would like to share our experience of management of an uncommon clavicular metastasis and illuminate the obscure mechanism of its scarcity.

**Case presentation:**

A 56-year-old female without any preknown systemic disease had suffered from a sole intermittent right shoulder pain without any other discomfort for 3 months. Physical examination performed at our orthopedic department showed tenderness over the right distal third of the clavicle with limited range-of-motion of the right shoulder. EGFR-mutated lung adenocarcinoma with metastasis over the right clavicle resulting in a pathological fracture was diagnosed according to the result of the incisional biopsy. Concurrent chemoradiation therapy accompanied with target therapy was performed. Eighteen months postoperatively, the clavicle pain was found to be subsided with stationary bony lesion under appropriate medication and palliative radiotherapy during the subsequent follow-up.

**Conclusions:**

The clavicle is an exceedingly unusual site with 2% of metastatic involvement of all type of tumors and only 1% among the population of carcinoma of lung due to its scanty red marrow and sparse vascular supply. Despite the unpleasant prognosis of clavicular metastasis from primary lung adenocarcinoma, promising quality of life is achievable under multidisciplinary management.

## Background

Lung carcinoma is the fourth most common form of the tumor (about 30 ~ 40%) spreading to the bone, behind breast (65 ~ 75%), prostate (65 ~ 75%), and thyroid cancer (about 60%) [1]. Among the patients of lung carcinoma, the most common metastatic sites are vertebrae, ribs, and pelvis [2]. By comparison, the clavicle is an extremely rare site of metastasis not only in lung cancer but of all types of tumors with only 1 ~ 2% involvement [3–5]. Owing to its scarcity, there is no firm guideline of the treatment among this population. With the consent of the patient, we herein present a case of lung adenocarcinoma located at the left upper lobe, who suffered from a right clavicular metastasis, for discussing the reason for its rarity and sharing our experience of management.

## Case presentation

**Fig. 1:**
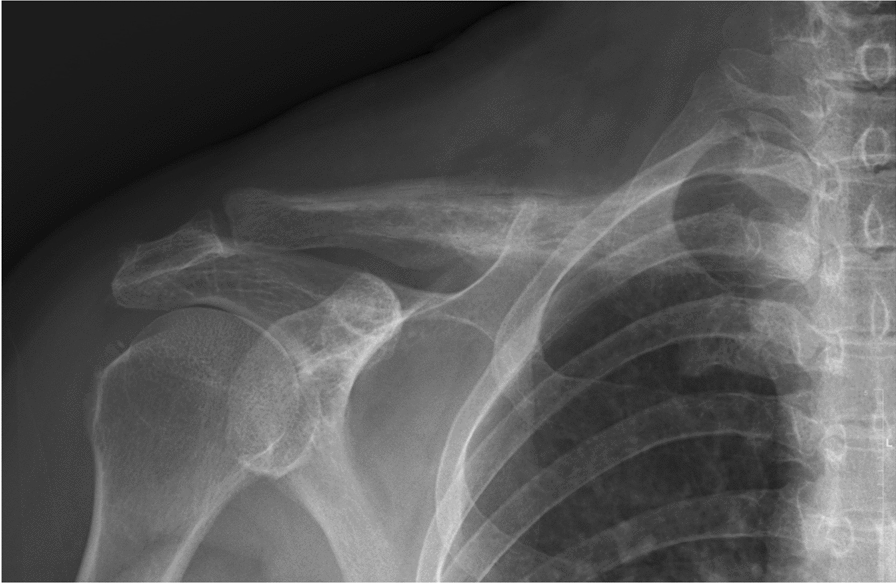
Plain radiograph of the right clavicle showed osteolytic moth-eaten appearance with osteosclerotic periosteal reaction

**Fig. 2:**
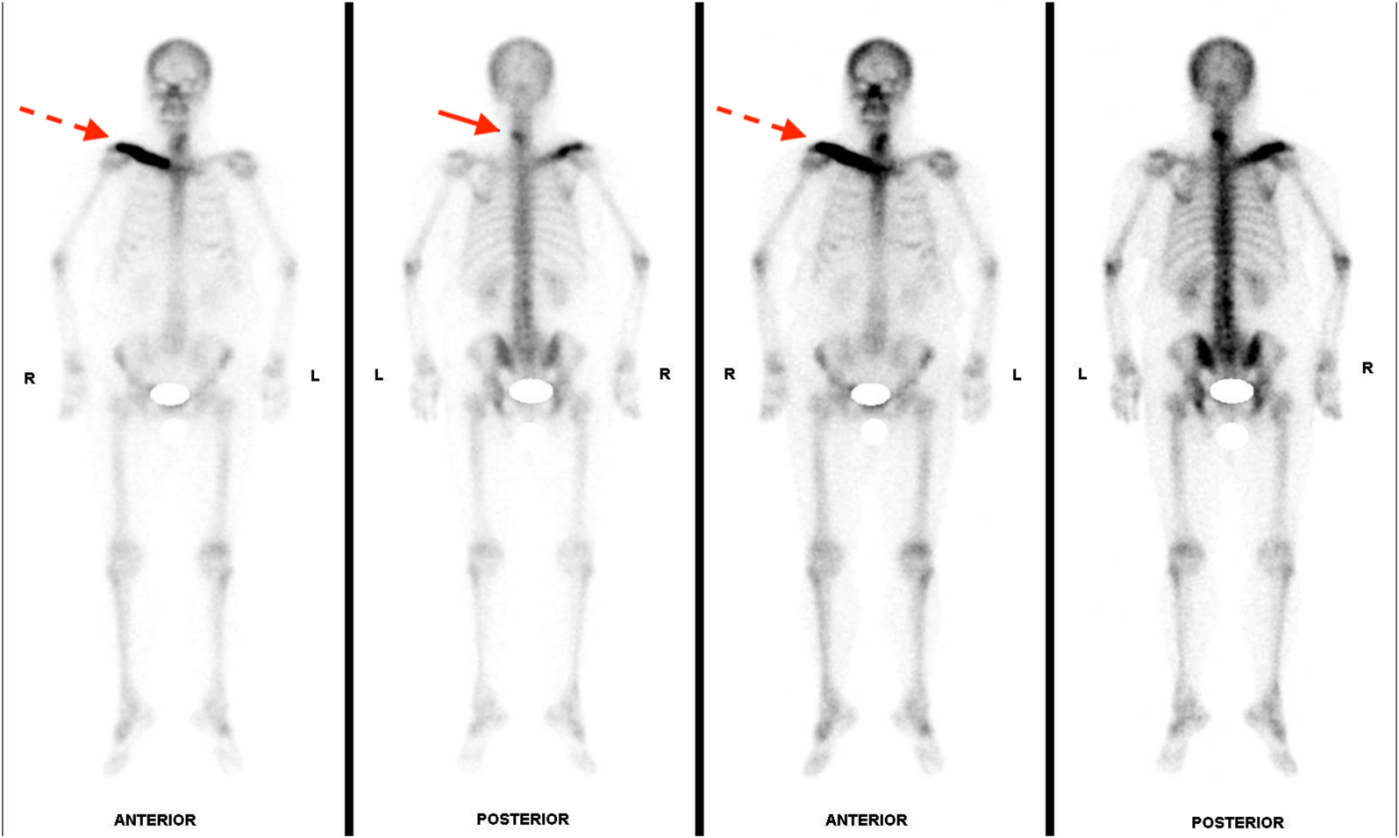
Whole-body bone scan revealed an intense linear uptake in the right clavicle and a focal hot spot in the cervical spine (Solid arrow: cervical vertebra; Dotted arrow: right clavicle)

A 56-year-old female without any preknown systemic disease presenting with an isolated intermittent right shoulder pain, especially at night, for about three months visited our orthopedic department for help. During the past three months, rheumatoid arthritis of the right shoulder was initially diagnosed and had been treated with medication at the other hospital, but in vain. Upon the physical examination at our department, tenderness over the right distal third of the clavicle accompanied with limited range-of-motion of the right shoulder was observed. Plain radiograph of right clavicle showed osteolytic moth-eaten appearance with osteosclerotic periosteal reaction (Fig. 1). Bone metastasis or osteomyelitis were firstly impressed. Subsequent whole-body bone scan revealed an intense linear uptake with osteosclerotic change over right clavicle and an osteolytic lesion with focal hot spot over cervical vertebra (Fig. 2). Thus, she was admitted to our ward for further evaluation and management.

**Fig. 3:**
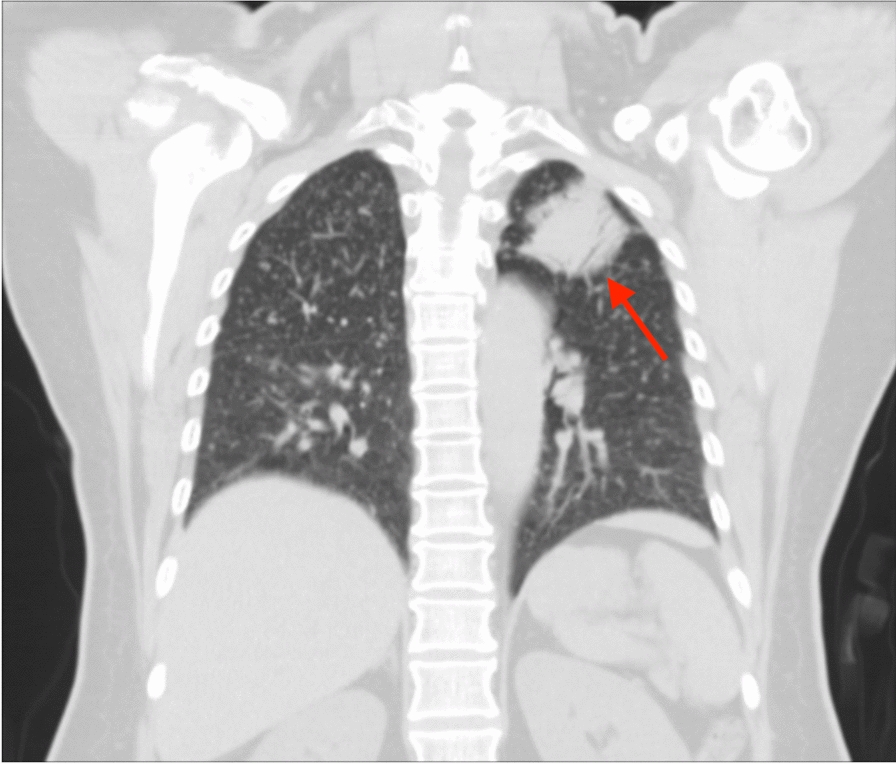
CT of the chest in lung window demonstrated a 4.4 cm mass (arrow) located at LUL of lung

**Fig. 4:**
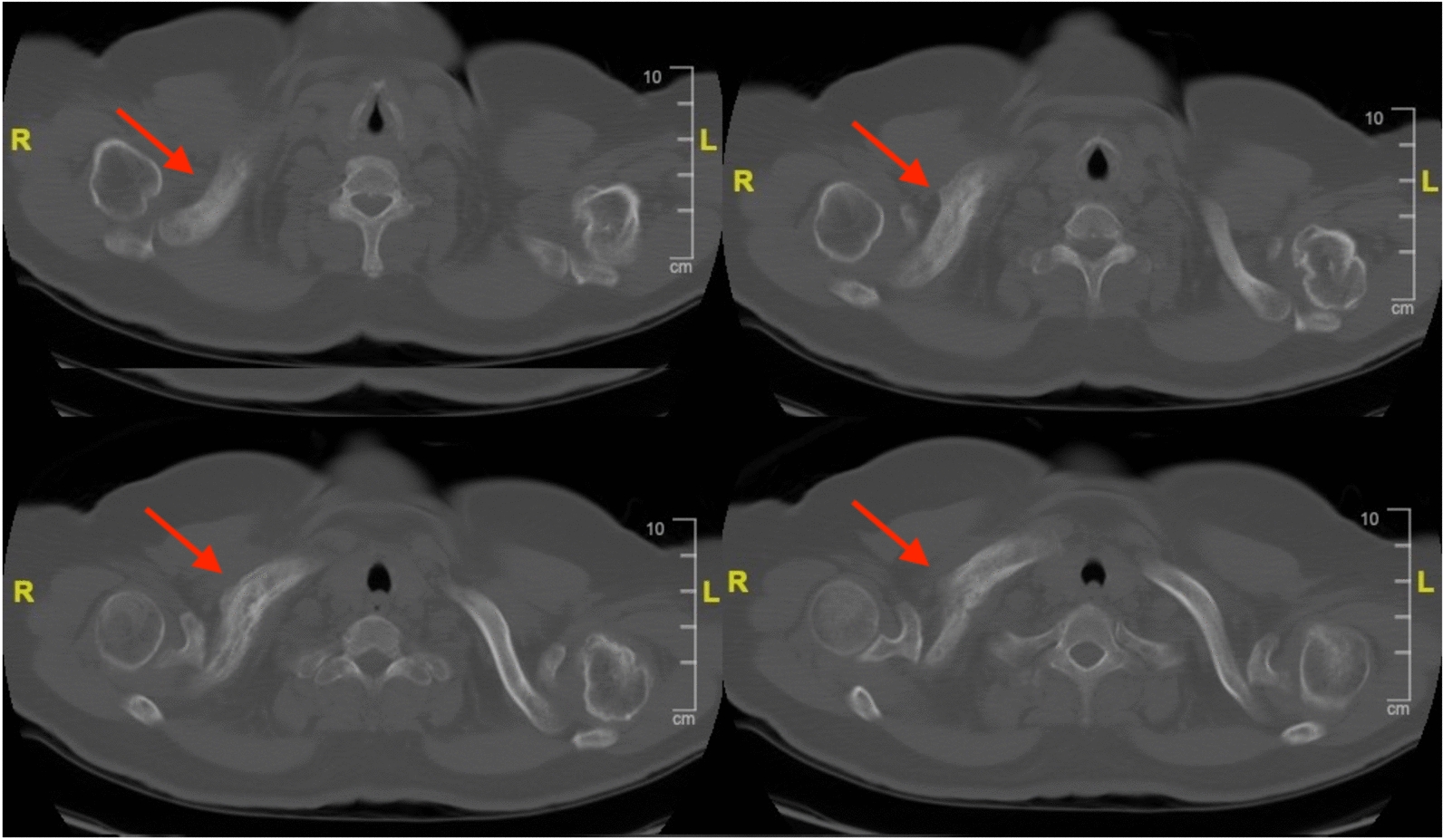
CT of chest in bone window disclosed heterogenous bone density with fracture and adjacent soft tissue lesion of right clavicle (Arrows: clavicle lesion)

**Fig. 5:**
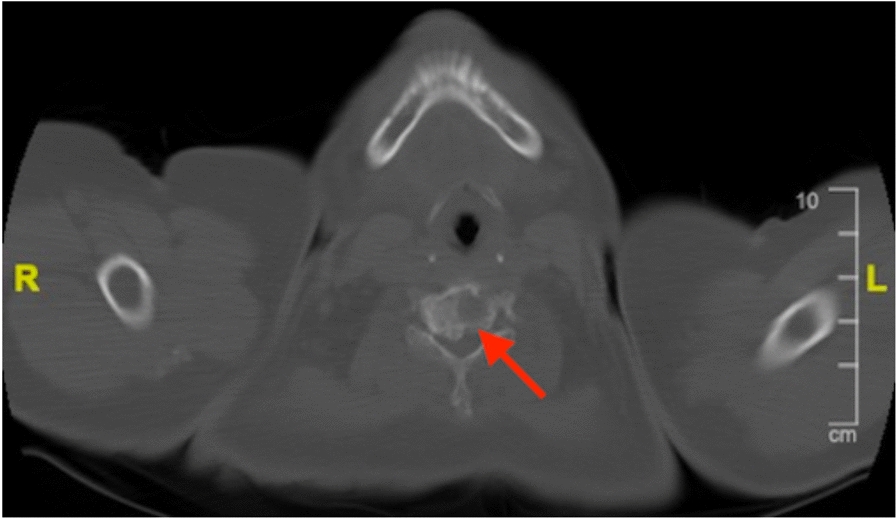
An osteolytic lesion (arrow) was observed over the vertebral body of C5

**Fig. 6:**
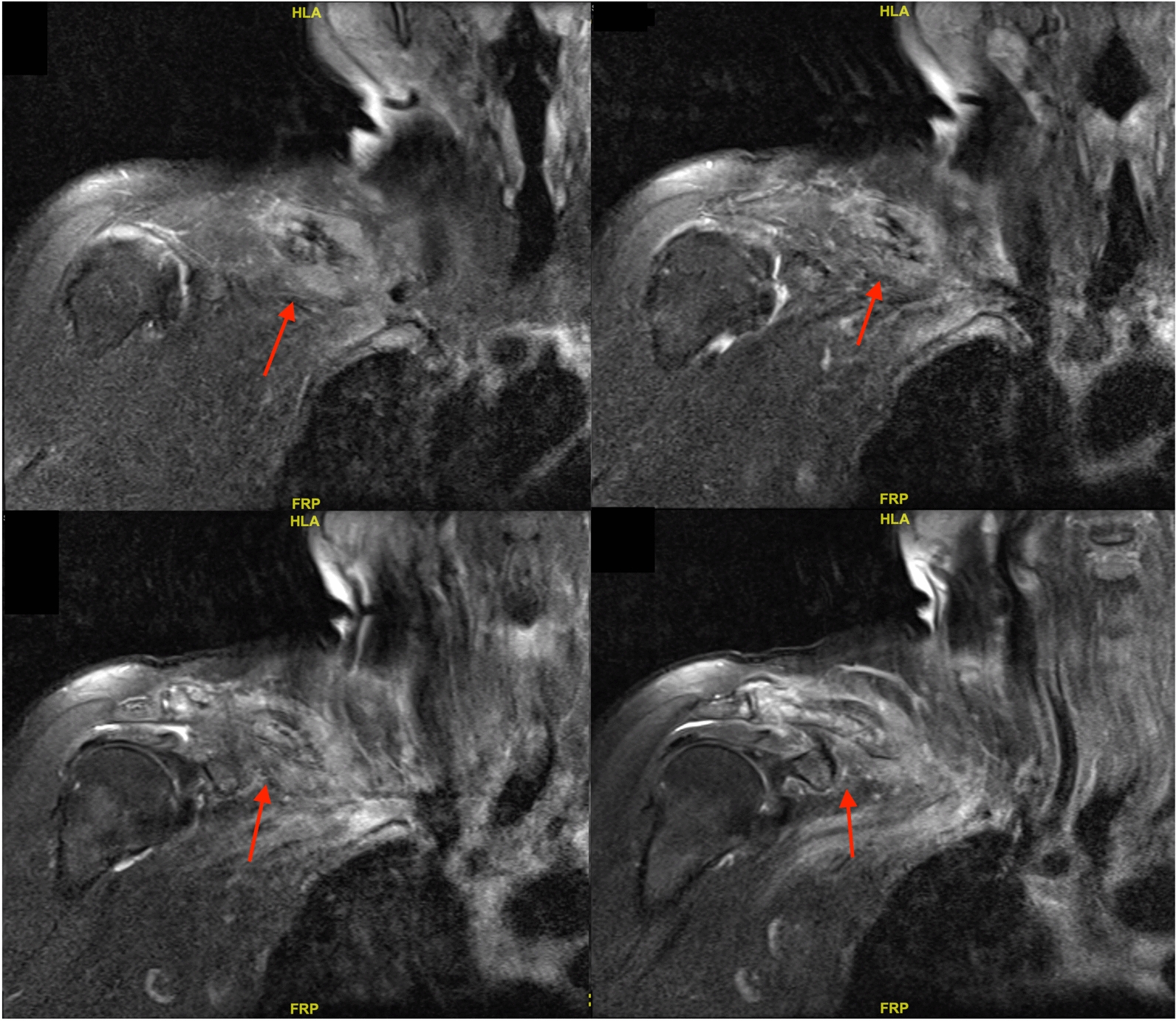
Magnetic resonance imaging (MRI) of right shoulder presented with a consistent result of an infiltrative mass lesion (9.3 cm*4.5 cm*3.3 cm, arrows) with a deconstruction of bone

After admission, we performed a list of work-up for tumor. Blood analysis showed elevated carcinoembryonic antigen (CEA) level without any other abnormality. Computed tomography (CT) of chest demonstrated a 4.4 cm mass over left upper lobe (LUL) of lung with miliary nodules over bilateral lungs and enlarged lymph nodes over bilateral hilar and left lower paratracheal regions (Fig. 3). Heterogeneous bone density with fracture and adjacent soft tissue lesion was observed over right clavicle with an osteolytic lesion over vertebral body of C5 (Figs. 4, 5). Lung cancer of LUL with bilateral lung metastasis and bone metastasis, staged cT4N3M1c according to AJCC 8th edition of cancer staging, was diagnosed. Magnetic resonance imaging (MRI) disclosed a consistent result of an infiltrative mass lesion (9.3 cm*4.5 cm*3.3 cm) with the deconstruction of bone, transcortical, and extraosseous soft tissue invasion arising from right clavicle (Fig. 6). With the consent of the patient, an incisional biopsy of the right clavicular lesion and a CT-guided biopsy of the tumor over LUL were performed step by step. After the operation, she had a stable postoperative course and was soon discharged home.

The specimen of the lesion over the right clavicle disclosed a microscopical picture of metastatic carcinoma with tumor cells immunohistochemically positive for TTF-1 and negative for p40, which was compatible with the results of stains of primary EGFR-mutated lung adenocarcinoma. MRI of the brain showed no imaging evidence of brain metastasis. Under the diagnosis of lung adenocarcinoma with bone metastasis, we conducted a series of multidisciplinary treatments for her. For the treatment of EGFR-mutated advanced non-small cell lung carcinoma (NSCLC), gefitinib (250 mg) was prescribed once a day. As for the bone metastasis, palliative radiotherapy (RT), total dose of 30 Gy in 10 fractions over the right clavicle and C5 in the period of 2 weeks, was administered with subcutaneous injection of denosumab (120 mg) once a month.

**Fig. 7:**
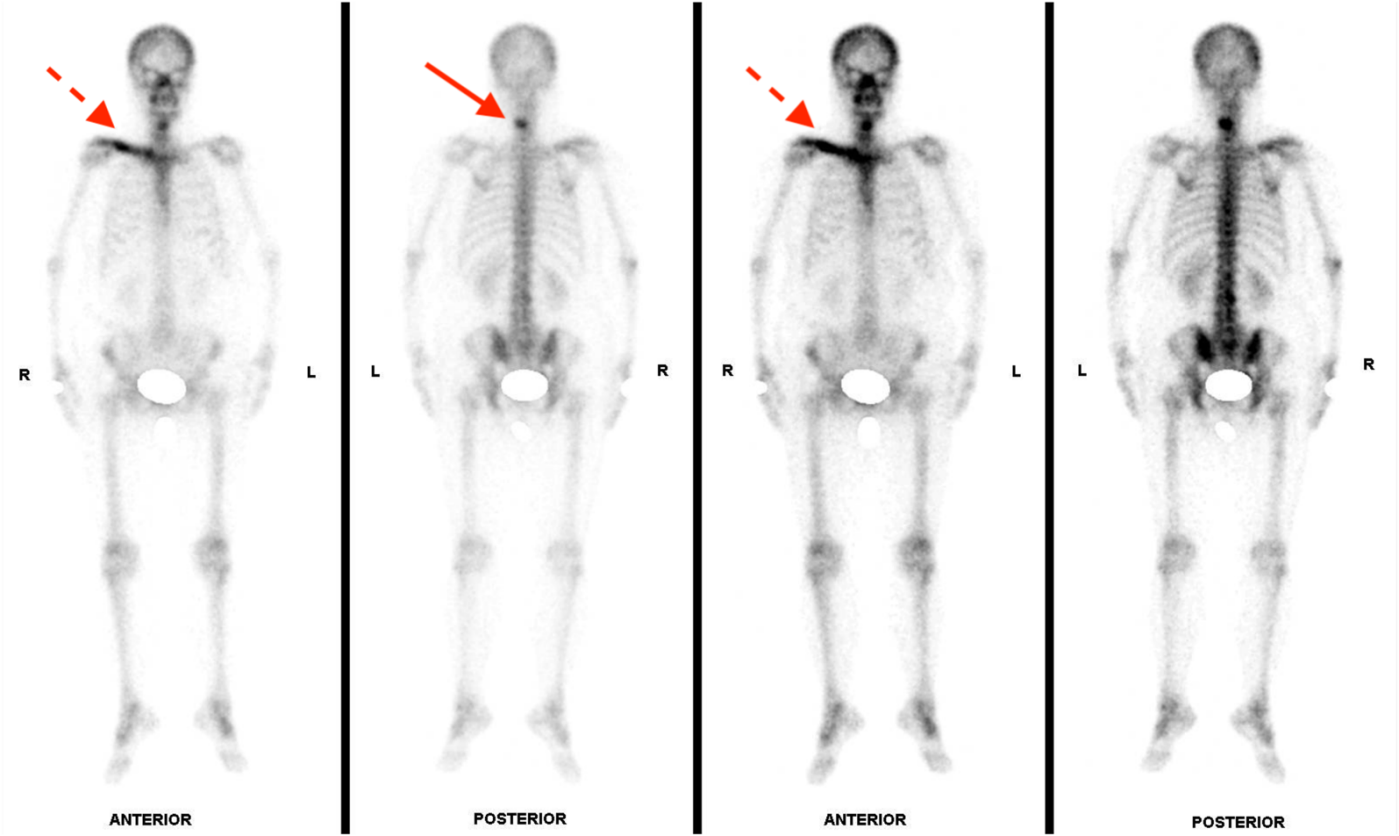
Four months postoperatively, whole-body bone scan revealed partial resolution of previous intense uptake over right clavicle and C5 with post-treatment response (Solid arrow: cervical vertebra; Dotted arrow: right clavicle)

**Fig. 8:**
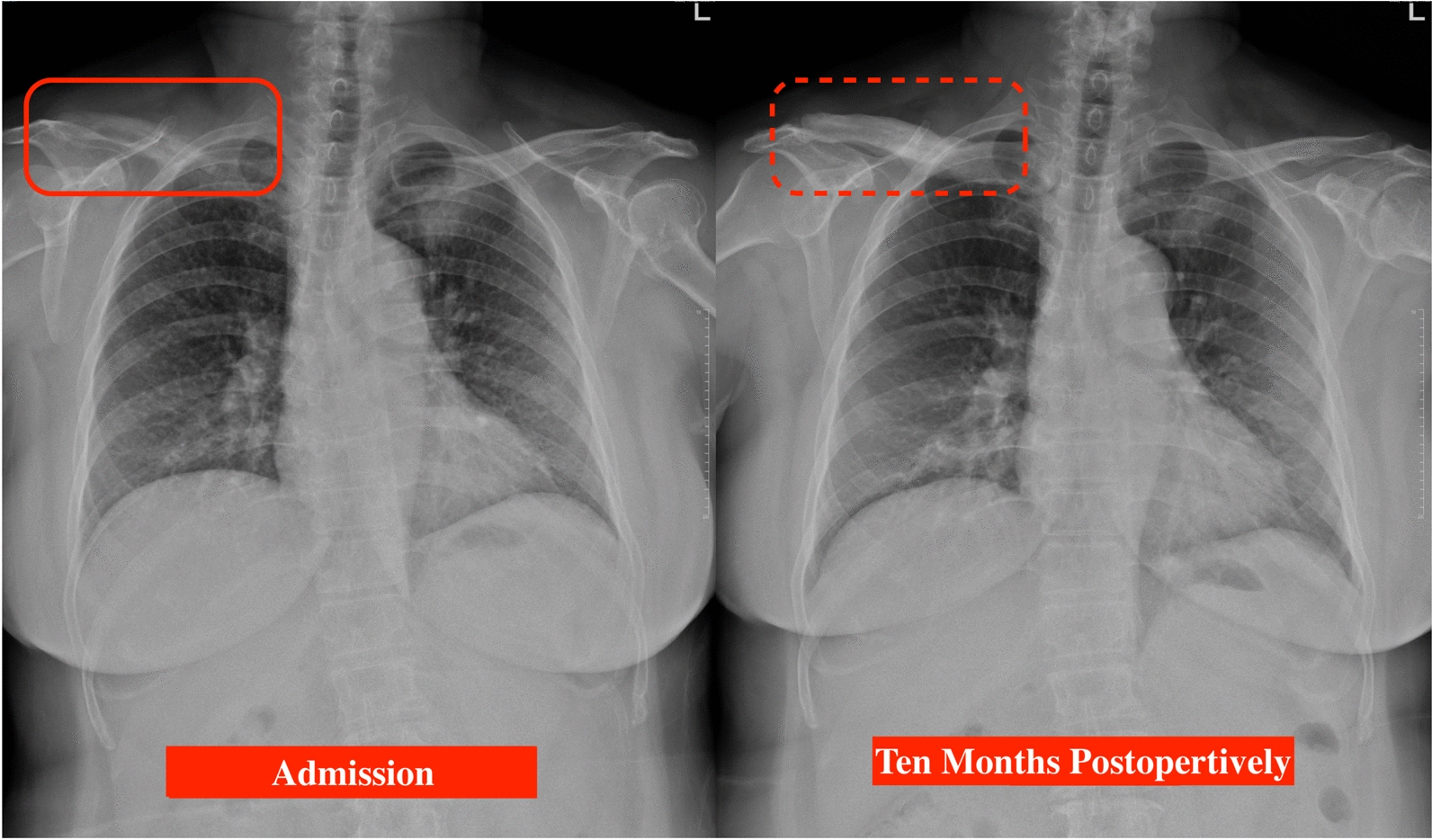
In comparison with the chest x-ray performed at the admission, the latest image presented with a focal sclerotic change of right clavicle with obviously increased bone density (Solid frame: admission; Dotted frame: the latest follow-up, 10-months postoperatively)

In the past 1 year postoperatively, right clavicular pain had been gradually subsided under the treatment of local RT and the usage of denosumab. Following whole-body bone scan showed partial resolution of previous intense uptake over the right clavicle and C5 with post-treatment response (Fig. 7). Follow-up of chest CT disclosed a progressively reduced size of tumor over LUL and stationary lesion over right clavicle, indicating partial response of target therapy. In comparison with the radiograph performed at the admission, the latest image presented a focal sclerotic change of right clavicle with obviously increased bone density (Fig. 8). Under multidisciplinary management of orthopedist, pulmonologist, and radiation oncologist, her clinical condition is now stationary without further progression. Regular outpatient follow-up will be arranged for closely monitoring the primary lung cancer and metastatic bone lesion.

## Discussion

Lung carcinoma is the fourth most common form of the tumor (about 30 ~ 40%) spreading to the bone, behind breast (65 ~ 75%), prostate (65 ~ 75%) and thyroid cancer (about 60%) [1]. Bone has been reported to be the second most common distant metastatic site of lung cancer (34%) and the most common one of NSCLC (34.3%) [6, 7]. However, traced back to the published literature, researchers declared the clavicle to be an exceedingly rare site of metastasis not only in lung cancer but of all types of tumor [3–5]. Thai et al. reported a retrospective analysis of 93 cases with 95 sites of bone metastasis to the humerus and shoulder girdle. There were only 2 cases of clavicular metastasis among 93 patients of all types of tumor [3]. Sugiura et al. retrospectively reviewed 118 cases of lung cancer with 318 sites of bone metastasis, in which the incidence of metastasis to the vertebra (42%), ribs (20%), and pelvis (18%) accounted for the majority. Metastases to clavicle was reported to occur in only 1% of patients [4]. Tsuya et al. demonstrated a retrospective investigation of 70 cases of non-small cell lung carcinoma accompanied with bone metastasis, which revealed a familiar result of dominant metastasis to the vertebra (50.0%) and rib (27.1%). There was even no case of clavicular metastasis reported in this population [5]. On the basis of published literature, the clavicle was considered to be a rare site of metastasis, which occurred in our case. This existing fact enlightened us to search for the obscure mechanism of the scarcity of this site of metastasis.

Metastasis is a series of processes including dissociation from the primary tumor, cell migration, access to the systemic circulation, escape of immunosurveillance, and eventually growing at the distant organs [8]. Bone metastasis, or to be precisely, bone marrow metastasis, is facilitated by the fenestrated structure of the red marrow sinusoid capillaries for transvascular migration of the tumor cells from the nutrient artery to the bone marrow and adhesive factors on tumor cell for adhesion to the stromal cells as well as the bone matrix. By disruption of the bone homeostasis, disseminated tumor cells in the bone marrow progressively covert the normal niches into metastatic lesions after overcoming immunosurveillance [9]. Owing to the characteristic mechanism, abundant amount of hematopoietically active red bone marrow and high blood perfusion of the metastatic area indeed play an indispensable role in the process of metastasis. The distribution of red bone marrow and the sufficiency of its blood supply perfectly demonstrated the reason why axial bones are much more commonly involved rather than the appendicular bones [4, 8–10].

Clavicle is seemed to meet the nature of insufficient red marrow and blood supply, which contributed to its rarity of metastasis. In terms of blood perfusion, Knudsen et al. declared three tiny branches of the subclavian artery including the suprascapular artery, the clavicular branch of the thoracoacromial artery, and the internal thoracic artery to be the primarily periosteal blood supply of the clavicle. No nutrient artery was observed [11]. Lack of its own main nutrient artery, the clavicle, unlike the most of other long bones, does not have an adequate medullary cavity to store the red bone marrow for nurturing the circulating tumor cells. As mentioned above, apart from the blood supply, the amount of hematopoietically active red bone marrow in the clavicle is relatively scanty to build a suitable niche for metastasis. In the 1980s, Cristy et al. published an analysis of calculating the regional distribution of active bone marrow at various ages via multiple methods of previous studies. Among all bones in our bodies, the percentage of active red marrow in the clavicle is the most impoverished one, with merely 0.7 ~ 1.0% at each stage of age [12]. In the 2000s, Caracappa et al. conducted an analysis of two methods of calculating red bone marrow mass via the radiation dose of computed tomography. Through these two methods, the calculated masses of red bone marrow in the clavicle are the second to last among the whole body, with only 16.7 g and 11.0 g respectively [13]. By comparison, in these two studies mentioned above, the bellwether of incidence of bone metastasis such as vertebra, rib, and pelvic all play the leading roles in not merely the percentage but the actual weight of red bone marrow as well [12, 13]. The result coincidentally corresponds to the observation of the review of Sugiura et al. that a high concentration of red bone marrow positively correlates to a high incidence of bone metastasis [4].

As for the management of the clavicular metastasis, palliative radiotherapy including single 8 Gy fraction and multiple fraction regimens such as 30 Gy in 10 fractions, are recommended for pain relief and maintain or ameliorate skeletal function [14, 15]. Denosumab, a human monoclonal antibody that specifically binds to receptor activator of NF-kB ligand which regulates the bone modeling, provided a tremendous impact on delaying the time of the first skeletal-related event and presented a promising overall survival, time to disease progression and safety profile [16, 17]. For its unpleasant outcome, improvement of quality of life should be the given priority for any therapeutic approaches in the management of bone metastasis.

## Conclusions

With its sparse vascular supply and scanty red marrow, the clavicle is an extremely rare site with only 2% of metastatic involvement of all types of tumors [3, 18]. Although the exceedingly low incidence of clavicular metastasis of primary lung adenocarcinoma foretells an unfortunate short-term median survival [19], the patient’s quality of life can be significantly improved with a favorable prognosis under multidisciplinary management.

## Data Availability

Not applicable.

## Abbreviations

CEA: Carcinoembryonic antigen
CT: Computed tomography
LUL: Left upper lobe
MRI: Magnetic resonance imaging
NSCLC: Non-small cell lung carcinoma
RT: Radiotherapy
